# A Novel Topology Control Approach to Maintain the Node Degree in Dynamic Wireless Sensor Networks

**DOI:** 10.3390/s140304672

**Published:** 2014-03-07

**Authors:** Yuanjiang Huang, José-Fernán Martínez, Vicente Hernández Díaz, Juana Sendra

**Affiliations:** Centro de Investigación en Tecnologías Software y Sistemas Multimedia para la Sostenibilidad (CITSEM), Campus Sur Universidad Politécnica de Madrid (UPM), 28031 Madrid, Spain; E-Mails: jfmartin@diatel.upm.es (J.-F.M.); vhernandez@diatel.upm.es (V.H.D.); jsendra@euitt.upm.es (J.S.)

**Keywords:** Wireless Sensor Networks, fuzzy-logic control, topology control, network connectivity

## Abstract

Topology control is an important technique to improve the connectivity and the reliability of Wireless Sensor Networks (WSNs) by means of adjusting the communication range of wireless sensor nodes. In this paper, a novel Fuzzy-logic Topology Control (FTC) is proposed to achieve any desired average node degree by adaptively changing communication range, thus improving the network connectivity, which is the main target of FTC. FTC is a fully localized control algorithm, and does not rely on location information of neighbors. Instead of designing membership functions and if-then rules for fuzzy-logic controller, FTC is constructed from the training data set to facilitate the design process. FTC is proved to be accurate, stable and has short settling time. In order to compare it with other representative localized algorithms (NONE, FLSS, k-Neighbor and LTRT), FTC is evaluated through extensive simulations. The simulation results show that: firstly, similar to k-Neighbor algorithm, FTC is the best to achieve the desired average node degree as node density varies; secondly, FTC is comparable to FLSS and k-Neighbor in terms of energy-efficiency, but is better than LTRT and NONE; thirdly, FTC has the lowest average maximum communication range than other algorithms, which indicates that the most energy-consuming node in the network consumes the lowest power.

## 1. Introduction

Topology control has been proposed as a technique to address many problems in networks by adding or deleting nodes/links according to certain algorithms/protocols, with the aim of obtaining expected network properties. In Wireless Sensor Networks (WSNs), the topology control is usually achieved by means of changing the communication range (equivalently, transmission power), scheduling sensor nodes to active/sleep mode, placing sensor nodes in specific positions, *etc.* Since energy availability is one of the most precious resources for sensor nodes, one of the goals of the topology control in WSNs is usually to reduce energy consumption, while at the same time preserving other fundamental properties, such as network connectivity, reliability, fault-tolerant, coverage, *etc*.

The node degree means the number of one-hop neighbors a node has. If all nodes in a network are still connected after removal of any *k* – 1 nodes, the network is called *k* – *connected* network. A network that is *k* – *connected* indicates that the node degree of each node is at least *k*, but the network is not necessary *k* – *connected* if the node degree for each node is at least *k*. The reasons considering the node degree as the goal of the topology control are the following. Firstly, the node degree suggests fault-tolerance capability of network. If a node has a higher node degree, it implies that there are more alternative paths to route the data when one or more neighbors fail. Secondly, if the node degree is the order of Θ(log *n*), where *n* is the total number of nodes in a network, the whole network is connected with high probability [1]. This result helps to design a localized algorithm, because each node in the network only has to take care of its own node degree. Thirdly, the node degree has an impact on the signal inference. Nodes experience lower contention when accessing a wireless channel if the node degree is lower. As a result, a tradeoff between network connectivity and link quality has to be taken into account.

In order to obtain the desired node degree, many algorithms are proposed to adjust sensor nodes' communication range to control the desired number of nodes in neighbor list, e.g., [2,3]. Because collecting global information is a time-consuming and energy-consuming process in large and distributed WSNs, these algorithms are usually localized, so they only rely on local and its one-hop neighbors' information. However, they also usually depend on calculating its neighbors' locations, e.g., through sensor nodes equipped with GPS, which is not always available in sensor nodes.

In this paper, we propose a localized fuzzy-logic approach, without the assumption that location information is needed, to adaptively control the communication range of each node in order to achieve the desired node degree. It is named Fuzzy-logic Topology Control, FTC for short. Fuzzy-logic is a very powerful control algorithm. Fuzzy-logic control has been proved to be a very successful control approach to many complex nonlinear systems [4,5]. It is relatively simple to convert knowledge of domain experts to control algorithms. Usually, the design of fuzzy-logic controllers starts with constructing the membership functions for linguistic input/output and if-then rules, such as [6,7]. Unfortunately, it is not easy to tune the parameters of membership functions and if-then rules for fuzzy-logic controllers to achieve desired performances when the system is very complicated or dynamic. For instance, if the number of nodes in a network increases or decreases because of node failures or adding new nodes, the membership functions may have to be re-designed, which would be a tedious work. Alternatively, we can also start from a training data set obtained from already acquired knowledge, such as the node degree distribution [8,9]. The fuzzy-logic controller can be constructed from the training data set, leaving the cumbersome process of tuning fuzzy controller parameters to learning algorithms. In this paper, we construct FTC from the training data set. More specifically, the training data set is derived from the mathematical description of the network. In addition, there is an integral controller outside fuzzy-logic controller to control the fuzzy-logic input.

To the best of our knowledge, this paper is the first that applies the fuzzy-logic controller based on a training data set to be used in topology control for WSNs. We study the impact of the integral controller parameters on network properties, and prove that the system is stable, accurate and has short settling time. In order to compare it with other representative localized algorithms (NONE, FLSS [2], k-Neighbor [10], LTRT [3]), FTC is evaluated by extensive simulations. The simulation results show that very similar to k-Neighbor algorithm, FTC is able to achieve desired average node degree even when node density varies, and FTC is better than NONE, FLSS and LTRT algorithms. Besides, in terms of energy consumption, FTC is comparable to that of FLSS and k-Neighbor, but saves more energy than NONE and LTRT.

The rest of this paper is organized as follows. An introduction of related works is provided in Section 2. Section 3 presents our proposed control algorithm FTC. We evaluate FTC by comparing it with other localized algorithms in Section 4. Section 5 concludes our work.

## 2. Related Works

Topology control for network connectivity issues has been widely studied. Latest surveys can be found in [11-14]. One of the fundamental problems in network is how to make the network connected or *k* – *connected*. For instance, if two networks are *k* – *connected*, the resultant network formed by joining them together is also a *k* – *connected* network if there are *k* – *vertex* disjoint edges connecting them [15]. The connectivity problem can be formalized as a linear programming (LP) problem [16–18]. Study [18] considers the special issue that some nodes' batteries are renewable. It presents the algorithm to optimize the power assignment by employing LP model. The connectivity problem can also be formalized as a Steiner tree or minimal spanning tree problem, e.g., [2,3,19–23].

Many works focus on study of the node degree *k*. One of the most interesting problems is the value of *k* to make the whole network connected. If all nodes in a network are uniformly deployed at random, there are some asymptotical results showing the condition when the entire network is connected with high probability as *n* → +∞, where *n* is the number of nodes in a network. There is a generic form: if *k* ≥ *c*_1_ log *n*, the network is connected with high probability; if *k* < *c*_2_ log *n*, the network is not connected with high probability. For instance, if *k* ≥ 0.5193 log *n*, the network is connected with high probability [24]. If *k* < 0.074 log *n*, the network is disconnected with probability one; if *k* ≥ 5.1774 log *n*, the network is connected with probability one [25]. Study [26] finds that if *k* ≤ 0.3043 log *n* the network is not connected with high probability; if *k* ≥ 0.5139 log *n*, then the network is connected with high probability. There is no evidence showing that *c*_1_ = *c*_2_. It is worth mentioning that once the network is connected with high probability, it is also immediately *k*–*connected* with high probability [24,27].

Previous results are asymptotical in nature, so they may not be able to be applied to real world scenarios easily. From a more practical perspective, it is necessary to propose topology control algorithms/protocols to make the network connected with high probability, while conserving as much energy as possible. However, the optimal solutions are usually not available. The problems, such as minimizing power consumption while maintaining a *k* – *connected* network [28]; minimizing number of links to obtain a 2 – *connected* network [29]; minimizing the number of node placement for *k* – *connected* [30]; minimizing the number of relay nodes for 2 – *connected* network [31,32], are NP-hard or NP-complete. Therefore, heuristic algorithms/protocols are proposed, such as [33], SPAN [34], CCP [35], PEAS [36], k-Neighbor [10], FGSS [2], FLSS [2], LTRT [3] and FTC, which is shown in this paper. Some of them are centralized algorithms, such as FGSS [2]. FGSS has proved that the maximum transmission radius among all nodes in the network is minimized. In WSNs, because the number of the nodes deployed in the field is very large, processing capability is relatively low, and power in each sensor nodes is very limited, a centralized algorithm is not very practical. Hence, localized and lightweight algorithms/protocols are expected for WSNs. Ref [33] proposes a greedy algorithm: it starts from a complete graph, and then reduces nodes to reach desirable connectivity, or starts from an empty graph and later connect edges until connectivity is reached, then it cuts off unnecessary nodes but maintains the same connectivity. In this paper, our proposed FTC is a localized approach based on fuzzy-logic theory and does not depend on neighbor location information. The results show that FTC is able to achieve comparable performances to FLSS and better than LTRT, but FLSS and LTRT both utilize the neighbor location information.

Control theory has been applied to WSNs. Ref [37] proposes a protocol that applies a control-theoretic approach to control packet reception ratio (PRR) directly. The receiver monitors all incoming packages and records the package transmission failures, and then computes the transmission power needed for next round. This information will be sent to the sender side. Some advanced control theories, such as fuzzy-logic control, are proposed to address various challenges in WSNs, e.g., energy optimization [38,39], clustering [40], and routing issues [41,42]. The one tackled in this paper is neither a routing, nor a network clustering problem. In general, routing or clustering algorithms are built on the basis that the network topology is well connected. We study a more fundamental problem from the network connectivity point of view. As presented in this paper, the fuzzy-logic system serves as an inference engine for each sensor node to modify its communication range. Unlike other fuzzy-logic control methods for WSNs, such as [6,7,38–43], this paper employs a training data set to design fuzzy-logic controller, rather than starting from designing membership functions and if-then rules. Our proposal is more flexible to deal with network dynamics, such as different node densities.

## 3. Fuzzy-Logic Topology Control (FTC)

In this section, we first present the design of Fuzzy-logic Topology Control (FTC), and then propose a protocol that can run on sensor nodes. We utilize topology control based on fuzzy-logic to achieve the desired node degree, in turn improving the network connectivity. Because WSNs are large-scale and distributed networks, the algorithm/protocol running in a sensor node is localized, only depending on the node itself and its one-hop neighbors' information.

### 3.1. WSNs Topology Control Using Fuzzy-Logic

In general, there are two ways to design the fuzzy-logic controller. The most commonly used approach is to design the membership functions and the if-then rules on the basis of understanding the system from domain experts. However, due to the complexity and dynamic of the system (such as WSNs), the design of the membership functions and if-then rules might not be easy. Without any (or with incomplete) knowledge about a system, tuning the parameters often takes very long time, e.g., the shape of the membership functions. Another way to design fuzzy controllers is to use the training data set obtained from extensive experiments or mathematical description if it exists. By means of applying some learning algorithms, the fuzzy controller can be learnt from training data set. In this paper, our FTC leverages the second approach.

#### 3.1.1. FTC Output and Input

**Figure 1.**
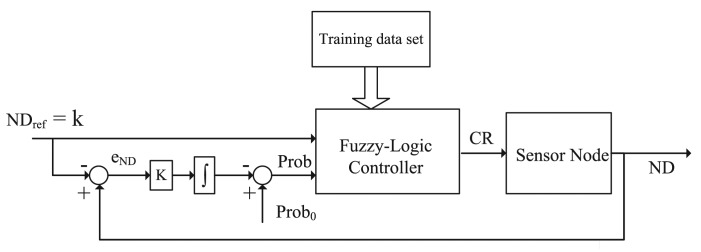
Fuzzy-logic topology control (FTC).

Figure 1 shows the system design of FTC. Adjusting the communication range (or equivalently transmission power) is a very common capability in many sensor nodes, e.g., Sun SPOT (Sun Small Programmable Object Technology) sensors. Hence, the output of FTC is the communication range (CR). Since the target is to reach a specific node degree (ND) for FTC, one of the inputs is the desired or reference ND, denoted by *ND_ref_* or *k*. Note that in this paper, we use *ND_ref_* and *k* interchangeably, because *ND_ref_* is used in control systems and *k* commonly represents the node degree in the graph theory. On the other hand, for a large and random network, the number of nodes within its communication range is unknown. Nevertheless, according to the network deployment strategy, the probability that a node has node degree *k* is known. For a random and uniform deployment, the probability that the node degree is at least bigger than *k* is given by Equation (1) (see [9]), where *r* is the node communication range, *n* is the total number of nodes in the field, node density $\rho =\frac{n}{A}$, and *A* is the area of the field. Therefore, the probability that a node has degree *k* is another fuzzy-logic controller input, denoted by *Prob*. In practice, *k* is an integer and communication range has an upper bound *r_max_*, so integer *k* > 0 and 0 ≤ *r* ≤ *r_max_*.

(1) $$P(ND\geq k)=f(k,r)=1-\sum_{N=0}^{k-1}\frac{{\left(\rho \pi r^{2}\right)}^{N}}{N!}e^{-\rho \pi r^{2}}$$

#### 3.1.2. FTC Learning Data Set

Provided a training data set from Equation (1), the fuzzy controller can be obtained through learning algorithms, such as neuro-adaptive learning algorithm. With the help of the Matlab “adaptive neuro-fuzzy inference system” (ANFIS) tool, FTC controller is not difficult to get. In ANFIS, the membership function parameters are automatically tuned through a backpropagation algorithm individually or in combination with a least square method. Once the data training is completed, the fuzzy inference system is available by simply using the function “evalfis” provided by Matlab. Hence, we concentrate on how to generate the training data set. As illustrated in Figure 1 and Equation (1), the inputs are *ND_ref_* and *Prob*, and the output is *CR*. Given *ρ, ND_ref_* ∈ {*k*_1_,*k*_2_,⋯ *k_m_*} and *CR* ∈ {*r*_1_, *r*_2_,⋯, *r_j_*}, *Prob* = *f*(*ND_ref_,CR*) can be calculated from Equation (1). The training data set ***T*** is a *s* × 3 matrix in the form of [*ND_ref_, Prob, CR*], where *s* = *m* · *j*. For instance, one item in the training data set could be [3, 0.9, 25]. It means that the CR is set to 25m if the probability that *ND_ref_* ≥ 3 is 0.9.

#### 3.1.3. *Prob*_0_ and K

Since *ND_ref_* is characterized by the probability, it is necessary to adjust the node degree if a node does not reach *ND_ref_*. For instance, if *CR* = 25 m can not lead to actual *ND* = *ND_ref_*, then next step is to adjust *Prob* to a higher value according to the node degree error *e_ND_*. As shown in Figure 1, there is an integral controller outside the fuzzy control to adaptively change *Prob*. From the control theory point of view, the system properties (e.g., steady state) are controlled by parameter *Prob*_0_ and *K*. If *e_ND_* is less than 0, *K* is configured to be half of its initial value. As a result, *CR* tends to increase faster than decrease. In Section 4.2, we will further discuss the impact of *Prob*_0_ and *K* by simulation.

### 3.2. FTC Protocol

According to the design of FTC in Section 3.1, corresponding FTC protocol is presented in this section, as shown in FTC protocol running for a generic node. Each node broadcasts the HELLO message at the maximum communication range *r_max_*, in order to collect neighbors' information. The communication range is modified accordingly based on the fuzzy-logic controller.

On the one hand, in this paper, we only consider undirected links (that is, there is a link between two nodes if and only if they are both in each other's communication range), because in practice many routing protocols assume that the link between two nodes are undirected. On the other hand, FTC is a localized control algorithm running at each node independently. As a result, the node degree changes over time. For instance, node *u* is the neighbor of *υ* at this round of communication range updating, but it is likely that in next round *u* is not the neighbor of *υ* due to the communication range of *u* and/or *υ* being altered. We will show in Section 4.2 that the system goes to steady state by appropriately defining the value of “rounds” in the FTC protocol.


FTC protocol (for a generic node *i*)**Require:**1:Training data set, ***T*** = [***k***, ***Prob***, ***CR***];2:Maximum communication range, *r_max_*;3:Reference node degree, *ND_ref_*;4:Initial Probability, *Prob*_0_;5:Initial *K, K*_0_;6:Number of rounds, *rounds*;**Ensure:**7:Obtain the fuzzy-logic control system by ANFIS, ANFIS(***T***);8:*CR_i_* ⇐ *r_max_*;9:*Prob* ⇐ *Prob*_0_;10:**while**
*rounds* > 0 **do**11: Broadcast HELLO message with current *CR_i_*;12: For messages received from other nodes, store the ID of its neighbors;13: Calculate the number of neighbors *ND* in the neighbor list;14: Calculate *e_ND_* = *ND* − *ND_ref_*;15: **if**
*e_ND_* < 0 **then**16:  *K* = *K*_0_;17: **else**18:   
$K=\frac{K_{0}}{2}$;19: **end if**20: *Prob* ⇐ *Prob* − *K* · *e_ND_*;21: *CR_i_* ⇐ *eval f is*(*ND_ref_, Prob*); %The output *CR_i_* can be calculated by *CR_i_* = *eval f is*(*k, Prob*)22: *rounds* ⇐ *rounds* − 123:**end while**


## 4. Simulation-Based Performance Evaluation

In this section, we evaluate our proposal FTC by using Matlab, comparing FTC with three representative localized topology control algorithms (FLSS, k-Neighbor, and LTRT), together with an algorithm without any control algorithms, called NONE in this paper. We will introduce them in Section 4.3.

### 4.1. Preliminaries

FTC is applicable to a network when all nodes are randomly and uniformly deployed, because the training data derived from Equation (1) assumes that all nodes are randomly and uniformly deployed. However, FTC can be applied to other deployment strategies as long as the node degree distribution is able to be obtained. In our simulations, nodes are uniformly deployed at random in a 100 × 100 m^2^ field. All nodes are stationary after the deployment. The maximum communication ranges, denoted as *r_max_*, for each node are the same, and *r_max_* = 30m. Each node is capable of adjusting its communication range within [0 *r_max_*]m. In this paper, we call one “round” simulation when all nodes finish the process of executing a topology control algorithm one time. Recall that *n* is the total number of nodes deployed in the field, and *k* is the desired node degree (namely, *ND_ref_* = *k*). In Section 4.4, in order to simulate different node densities, the number of nodes deployed in the area varies from 50 to 90 nodes. We randomly generate 50 networks for each specific number of nodes. For each network, we run different algorithms separately. The results are the average of 50 deployments.

### 4.2. Analysis of FTC Properties

**Figure 2.**
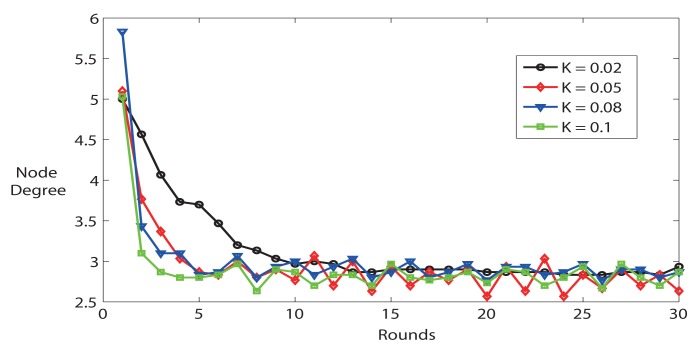
Average node degree with different K (*Prob*_0_ = 0.8, n = 60, k = 3).

As we mentioned in Section 3.1, FTC is affected by two parameters (*Prob*_0_ and *K*) outside the fuzzy-logic controller. The simulator provides average node degree calculated by the sum of node degree for all nodes divided by the number of nodes in the network. Figure 2 shows the impact of *K* on network settling time, accuracy and stability. Firstly, the average node degree quickly converges to its steady state value (after about 10 rounds). Secondly, the average node degree converges to the reference node degree *k* = 3. A lower *K* results in a more stable system, but longer convergence time. For instance, *K* = 0.02 is more stable than *K* = 0.1, but *K* = 0.02 takes longer time to reach steady state. Thirdly, the system is stable, but the system demonstrates heavier oscillation behavior when *K* is bigger.

**Figure 3.**
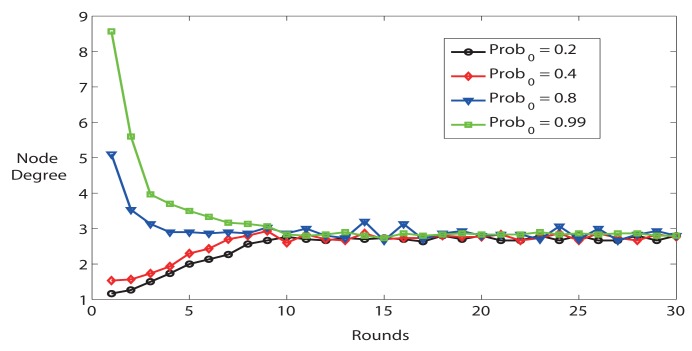
Average node degree with different *Prob*_0_ (K = 0.02, n = 60, k = 3).

Figure 3 illustrates different features at the first several rounds. If *Prob*_0_ is higher at the beginning, the nodes are more likely to be connected than *Prob*_0_ is lower. Nevertheless, the average node degree goes to stable state after 10 rounds as well. As a result, *Prob*_0_ has an impact on the initial status of network.

In short, the simulation results illustrate that *K* has an important influence on network settling time, accuracy and stability. In the following simulations, we choose *K* = 0.02, *Prob*_0_ = 0.8 and *rounds* = 15 to configure the parameters described in FTC protocol.

### 4.3. NONE, FLSS, k-Neighbor, LTRT

In this section, we briefly introduce four algorithms we have compared FTC with. More details can be found in the corresponding references.

- NONE: once all sensor nodes are deployed in the field, each node configures its communication range to the maximum communication range, and the communication range does not change during the simulation. NONE generates the most connected network, thus gives the upper bound of network connectivity. NONE algorithm is used to simulate the case that there is no topology control applied to WSNs.
- Fault-tolerant Local Spanning Subgraph (FLSS) [2]: each node runs at their maximum communication range to collect neighbors' information, e.g., IDs, locations. Based on this information, each node first sorts all edges in an ascending order, and then only selects edges according to the order if it is not *k* – *connected* to it. Maybe some edges are directed, but it is optional to only consider bi-directional edges by deleting directed edges, or turning directed edges into bi-directional edges.
- k-Neighbor algorithm [10]: each node first runs at its maximum communication range as well, in order to collect neighbor IDs and location information. Its final communication range is set to the distance between itself and its *k* – *th* nearest neighbor. One of the important features of k-Neighbor is that the algorithm is asynchronous. Each node starts running its topology algorithm at a random time. However, the difference between nodes wake up is bounded by a known constant Δ. By the time 4Δ+2*d*+*τ*, all nodes terminate setting the communication range, where *d* is the time to obtain a specific contention free wireless with probability P, and *τ* is the upper bound of processing time for sorting neighbors. In the circumstances that the number of neighbors is less than *k*, k-Neighbor algorithm modifies its communication range to the distance to the farthest neighbor.
- Local Minimum Spanning Tree (LTRT) [3]: LTRT performs local spanning tree algorithm *k* times. Given a network *G*(*V*, *E*), where *V* is the set of nodes, *E* is the set of links. LTRT calculates one of its spanning trees *T*(*V, Ê*_1_), and then removes all links in *Ê*_1_ from *E*. The resulting network is denoted as *G*(*V*, *E* – *Ê*_1_). Next, the same process is conducted on *G*(*V*, *E* – *Ê*_1_). After *k* times, the final network is formed by combining all trees together, that is *G*(*V*, *Ê*_1_ + *Ê*_2_ + ⋯ + *Ê_k_*). The final communication range of each node is the maximum communication range in *G*(*V*, *Ê*_1_ + *Ê*_2_ + ⋯ + *Ê_k_*).

FLSS, k-Neighbor, and LTRT share some common features: they are all localized algorithms; they all need location information of neighbors; they all aim at finding out *k* neighbors. There is an important difference among them, though: FLSS and LTRT have been proven to be able to preserve the network to be *k* – *connected* if the original network is at least *k* – *connected*. On the contrary, k-Neighbor algorithm only proves that the network is 1 – *connected* with high probability if *k* is the order of Θ (log *n*). Like FLSS, k-Neighbor and LTRT, FTC proposed in this paper is also a fully localized algorithm to find *k* neighbors, but FTC does not need location information. Similar to k-Neighbor, FTC cannot guarantee that the network is 100% connected either, but FTC can achieve the desired *k*, and therefore FTC can make the network connected with high probability if choosing *k* appropriately.

### 4.4. Comparison and Discussion

**Figure 4.**
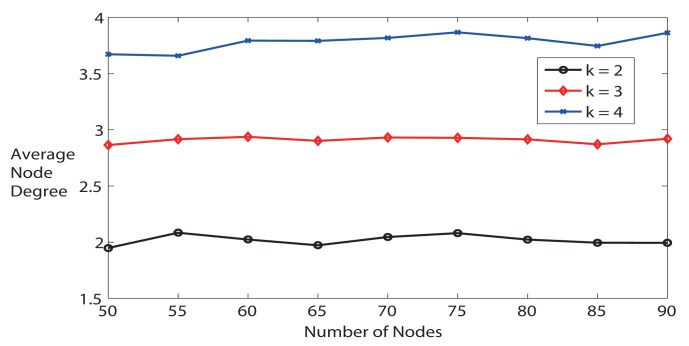
Different desired node degree k.

First, we evaluate whether FTC is able to trace the *ND_ref_* or *k*. As illustrated in Figure 4, the average node degree is very close to the desired node degree when *k* = 2 as the number of nodes varies from 50 to 90. When *k* is bigger, the average node degree is slightly lower than expected, because it is less likely to get more neighbors. For instance, with the same communication range, the probability of having 4 neighbors is obviously lower than having only 2 neighbors. In addition, the maximum communication range is limited. We can conclude from Figure 4 that FTC is able to achieve desired average node degree *k* without knowing the locations of neighbors. Later, we will show that only k-Neighbor algorithm can achieve desired node degree as FTC, but k-Neighbor requires location information of neighbors.

**Figure 5.**
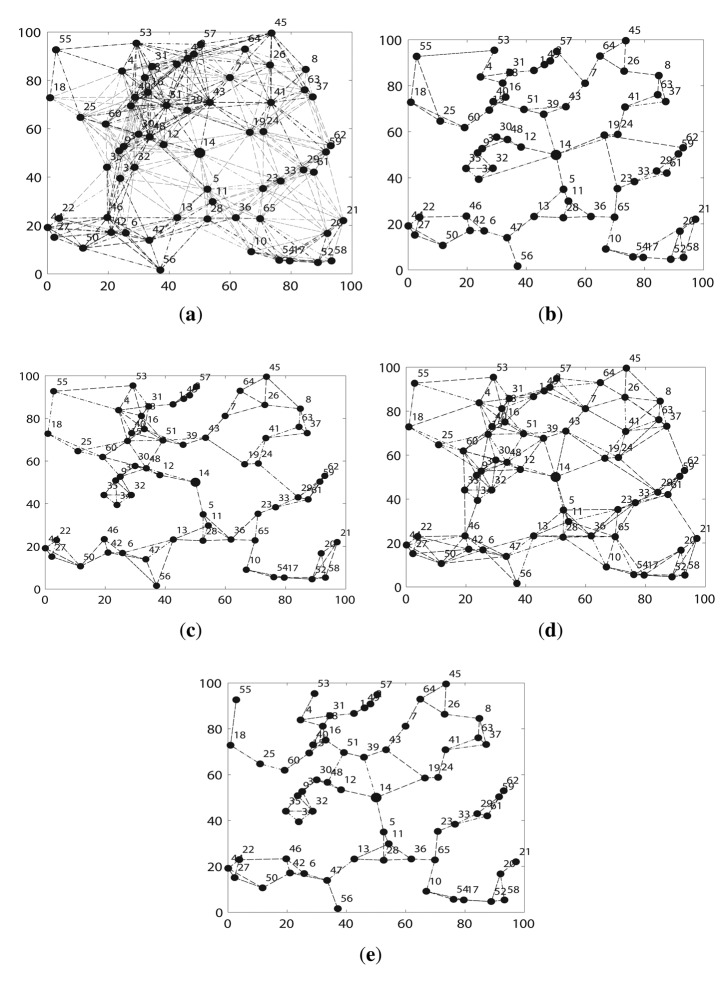
Example of resultant topology (n = 65, k = 3). (**a**) NONE; (**b**) k-Neighbor; (**c**) FLSS; (**d**) LTRT; (**e**) FT

Second, we compare FTC with other algorithms. Figure 5 shows an instance of the topology after running different topology algorithms on the same network when *k* = 3 and *n* = 65. Figure 5 a indicates that the network is very well connected, because each node runs at the maximum communication range, but it also causes severe signal inferences in the network, as well as too much energy consumption. LTRT in Figure 5d shows the second most connected network. FLSS in Figure 5c, k-Neighbor in Figure 5b and FTC in Figure 5e are quite similar. They preserve network connectivity with much lower amount of links between nodes, which means that much less signal inferences are introduced. Figure 5 implies that FLSS, k-Neighbor and FTC demonstrate a better tradeoff between network connectivity and link quality. FLSS may produce directional links. We only plot the undirected links, so the resulting network running FLSS shown in 5c is not a 3 – *connected* network, the original network shown in Figure 5a is at least a 3 – *connected* network though.

**Figure 6.**
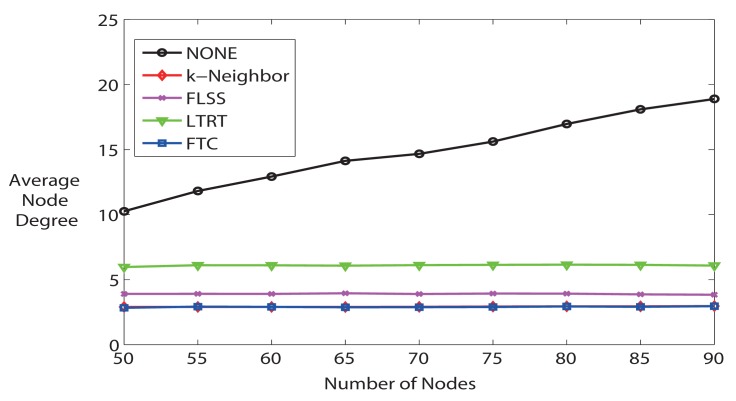
Average node degree (k = 3).

Third, we further study the average node degree. Figure 6 illustrates the average node degree for all algorithms when the number of nodes varies from 50 to 90. The average node degree increases when the number of nodes increases if running NONE algorithm, but other algorithms are able to keep the node degree at a much lower level. Particularly, FTC and k-Neighbor are not distinguishable in Figure 6, and they are the best algorithms that are able to trace *k*. k-Neighbor is able to get the exact *k*, because it calculates where the *k* – *th* nearest neighbor is, based on already known distance information. FTC, on the contrary, is able to reach the desired *k* (see also Figure 4), but without knowing the neighbor locations. LTRT's node degree is the second higher. As shown in previous Figure 5d, LTRT shows its superiority over FTC, FLSS, and k-Neighbors concerning the network connectivity.

**Figure 7.**
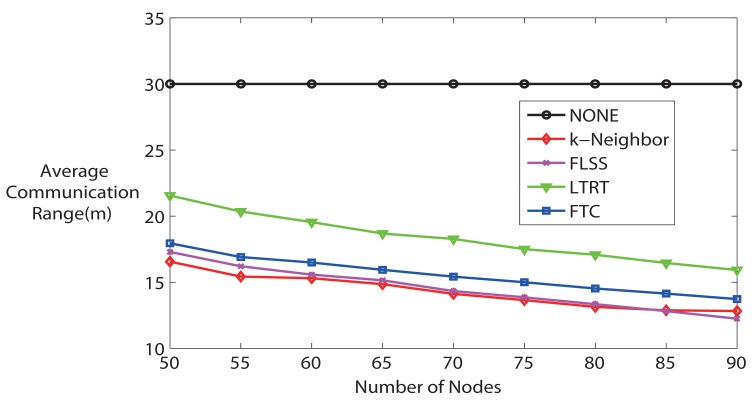
Average communication range (k = 3).

Moreover, it is worth noting that the communication range is proportional to the energy consumption of wireless sensor nodes. Therefore, a lower average communication range implies a lower energy consumption, which is a significant performance when designing WSNs. Figure 7 shows the average communication range. Recall that the maximum communication range of each node is 30 m in the simulation. On the one hand, apart from NONE algorithm (because the communication range of each node is fixed at maximum), other algorithms' average communication range decreases along with the number of nodes in the network increasing, because higher node density indicates that it is more likely to have the desired node degree with lower communication range. With the number of hops increasing, the energy consumption due to wireless transmission decreases, but the energy consumption due to forwarding messages increases. Overall, though, the energy is still saved, because the energy spent on wireless transmission is orders of magnitude higher than that spent on computation. So, we can conclude that energy consumption is lower if the node density is high for all algorithms (except NONE). On the other hand, from Figure 7, it can be seen that in terms of energy consumption, NONE is the worst, and LTRT is the second worst. FTC, FLSS and k-Neighbors performance is very close to each other, but it is better than NONE and LTRT.

**Figure 8.**
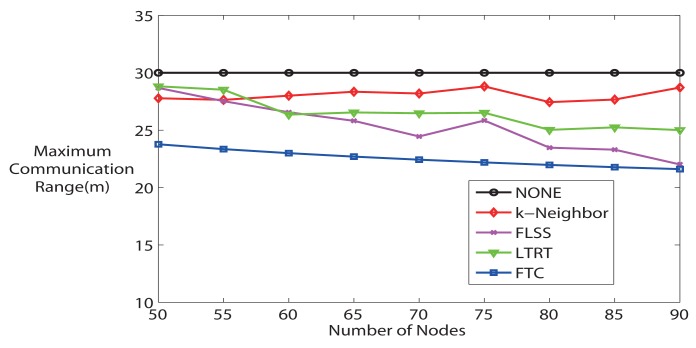
Average maximum communication range (k = 3).

**Figure 9.**
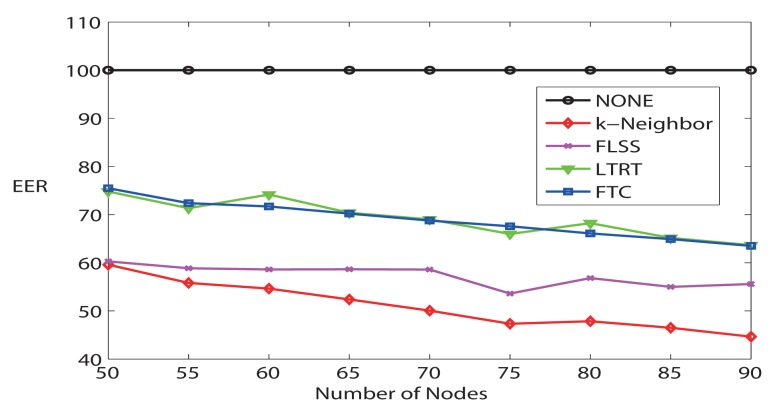
Energy expended ratio (*EER*) (k = 3).

Finally, Figure 8 demonstrates the average maximum communication range. FTC maintains the lowest maximum communication range comparing it with other algorithms, which means that the most energy-consuming node in the network consumes the lowest power. Furthermore, Figure 9 evaluates the Energy Expended Ratio (EER) (define $\text{EER}=100\times \frac{CR_{\text{avg}}}{CR_{\text{max}}}\%$) [28]. EER is a parameter wanted to be as small as possible. NONE is the highest; FTC is very similar to LTRT; while k-Neighbor and FLSS are relatively lower. FLSS claims that it has achieved near optimal performance.

## 5. Conclusions and Future Works

In this paper, we address the connectivity problem in WSNs. More specifically, we proposed a novel localized adaptive fuzzy-logic topology control algorithm, called FTC, to control the communication range of sensor nodes in WSNs, with the purpose of achieving the desired node degree (namely the number of one-hop neighbors a node has), therefore in turn improving the network connectivity. Unlike other ways to design fuzzy-logic control system, the fuzzy-logic controller of FTC is constructed from a training data set. One of the great advantages of this approach is that it is easier to design a feasible controller without complicated parameter adjustment for the fuzzy-logic controller, especially when the network is highly dynamic.

FTC has been compared with four representative localized algorithms: NONE, FLSS, k-neighbors and LTRT. The simulation results show that FTC is able to trace the desired node degree as k-Neighbor algorithm which is based on the locations of neighbors, but FTC does not depend on location information. On the contrary, NONE, FLSS, and LTRT are unable to achieve the desired node degree. The average communicating range of FTC is very close to FLSS and k-Neighbor, but lower than NONE and LTRT. It implies that the energy consumption of FTC is lower than that of NONE and LTRT. In addition, the average maximum communication of FTC is the lowest, which means that the highest energy-consuming node in the network running FTC protocol consumes the lowest power than running other algorithms. The Energy Expended Ratio (EER) of FTC is very close to LTRT, but higher than FLSS and k-Neighbor. In summary, without knowing the neighbor location information, our localized FTC shows the capability of achieving desired node degree, maintaining network connectivity, and it is energy-efficient.

In this paper, all nodes are stationary after they are deployed. In our future works, we will take into account the mobility of sensor nodes. Furthermore, our validations are based on computer simulations. In order to validate FTC protocol, real experiments will be carried out on our Sun SPOT sensor platform in the near future. The node deployment is very unlikely to be an ideal random and uniform distribution, so we will calculate all sensor nodes' locations through computer simulation prior to deploying them in the field.
